# Comparing the Emergency Care of Iranian and Afghan Patients During the COVID-19 Pandemic

**DOI:** 10.34172/aim.2024.22

**Published:** 2024-03-01

**Authors:** Milad Ahmadi Gohari, Maryam Chegeni‬‬, Mohammad Hossein Mehrolhassani, Ali Akbar Haghdoost, Moghaddameh Mirzaee

**Affiliations:** ^1^Modeling in Health Research Center, Institute for Futures Studies in Health, Kerman University of Medical Sciences, Kerman, Iran; ^2^Department of Public Health, Khomein University of Medical Sciences, Khomein, Iran; ^3^Health Services Management Research Center, Institute for Futures Studies in Health, Kerman University of Medical Sciences, Kerman, Iran; ^4^Department of Biostatistics and Epidemiology, School of Public Health, Kerman University of Medical Sciences, Kerman, Iran

**Keywords:** Afghan immigrants, COVID-19, Iran, Trauma

## Abstract

**Background:**

This study investigated the quality of inpatient care provided to Afghan immigrants in Iran during the COVID-19 pandemic (February 2019 to March 2021). For this purpose, the services received by Afghan immigrants were compared with those received by Iranian citizens.

**Methods:**

Two emergency services (traumas with 8080 victims and 8,686 patients hospitalized with severe COVID-19 infection) were taken into consideration. The records of all patients, including the Afghan immigrants, in two referral hospitals in Kerman were reviewed, and the main variables were the length of hospitalization (LoH), intensive care unit (ICU) admission rate, and death rate. Quantile regression, multiple logistic regression, and Cox regression were used to analyze the data.

**Results:**

The median and interquartile range of LoH for Afghan and Iranian nationals admitted due to traumas were 3.0±4.0 and 2.0±4.0, respectively (*P*<0.01). Moreover, the chance of Afghan nationals being admitted to the ICU (38%, odds ratio=1.38; 95% confidence interval [CI]=1.12; 1.69) and the hazard of death (60%, hazard rate=1.60; 95% CI=1.03; 2.49) were higher compared to Iranian nationals, which is statistically significant. However, no significant differences were observed between the COVID-19 patients from the two nationalities in terms of the median LoH, the odds of being admitted to the ICU, and the hazard of death due to COVID-19.

**Conclusion:**

Afghan nationals admitted to the hospital due to traumas were more likely to be admitted to ICUs or die compared to Iranian citizens. It seems that Afghan patients who had traumas went to the hospitals with more serious injuries. There was no difference between Afghan and Iranian patients in terms of COVID-19 consequences. Following the findings of this study, it seems that justice in treatment has been fully established for Afghan patients in Iran.

## Introduction

 Afghanistan has one of the largest numbers of refugees in the world.^1^ During recent years, this country has been involved in civil and/or foreign wars almost continuously.^1^ Official statistics show that Iran and Pakistan are the destinations for more than 90% of Afghan immigrants.^1^ During the past four decades, Iran has welcomed and sheltered Afghan immigrants with open arms, placing it among the top ten countries that host refugees worldwide.^2^ According to the latest statistics released by the Iranian government, at least 780 thousand Afghans live in Iran as refugees ^3^. In addition, it is estimated that around 2.1 million undocumented Afghans and nearly 600 000 Afghan passport holders also reside in Iran.^3^ Since the developments and the critical situation in Afghanistan created in August 2021, the number of Afghans who have immigrated to Iran has increased sharply. Various estimates indicate that 0.5‒1 million Afghans have recently fled Afghanistan and come to Iran.^3^ Almost 96% of Afghan refugees live in urban areas, and the rest live in about 20 refugee settlements across the country.^3^ The provinces of Tehran, Mashhad, Isfahan, and Kerman are the most frequent destinations for Afghan immigrants. Official statistics represent that Afghan immigrants account for 8‒10% of the population of Kerman Province. About 12% of the refugee population lives in guest cities located in Kerman and Yazd provinces.^4^

 The health status of immigrants and refugees should be important for the officials of the host country,^5^ as the protection and health of the host communities depend on the health of the newcomers. Studies have shown that refugees and immigrants have acute mental health problems, especially depression and stress.^6,7^ Even though they need more treatment and healthcare, many reasons, among which are linguistic and cultural differences, organizational discrimination, poverty, illiteracy, and fear of reporting their immigration status to law enforcement, restrict their use of health services in destination countries. Thus, the immigrants and refugees are more vulnerable than the citizens of the host country.^7^

 The health of Afghan immigrants and nationals in Iran has always been a concern of politicians as a principle of human rights. Therefore, the Iranian government has made all basic medical care free for immigrants. In addition, in February 2021, with the start of the seventh round of national public health insurance in the country, vulnerable refugees who were eligible were notified by the United Nations High Commissioner for Refugees (UNHCR) to receive insurance booklets. The insurance service allows refugees, such as Iranian nationals, to access affordable secondary and tertiary healthcare through more than 1000 public hospitals across the country.^8^

 Health insurance services are provided to immigrants who are either registered as refugees in UNHCR or have a legal residence permit. Immigrants who have entered Iran illegally cannot apply for health insurance, but they can usually be treated in private or charitable medical clinics, for which they must assume all related costs.^9,10^ Although the government of Iran has attempted to provide healthcare and treatment services for immigrants, there are still obstacles, such as low literacy and poverty among immigrants and a lack of insurance coverage due to illegal immigration.

 COVID-19 was undoubtedly one of the most challenging health and treatment problems of the current century.^11^ This disease has affected various aspects of people’s lives in countries worldwide. According to official statistics, until December 5, 2023, 698.75 million people have been infected with this disease worldwide.^12^ Despite adopting numerous policies to prevent the transmission of COVID-19 across the country,^13,14^ Iran is still not immune from this disease. Until December 5, 2023, this disease infected about 7.62 million people and resulted in the deaths of about 146.7 thousand people.^12^ Estimates in Iran demonstrate that this disease has caused many additional deaths in the country.^15^ During COVID-19 peaks, hospitals in Iran, similar to other countries, were full of patients, and it was difficult to admit and treat patients. It was also the human duty of the health and medical staff to provide services to Afghan immigrants and other citizens.

 According to the official statistics in Iran in 2021 (1400-Hijri Shamsi), 16 778 and 317 120 people were killed and injured in driving accidents, respectively.^16^ During the last 10 years, accidents have caused about 153 000 deaths in Iran.^12,16^ Accidents are among the most important reasons for referring patients due to trauma. Therefore, casualties caused by driving accidents are considered one of the main concerns of Iranian health officials and policymakers.

 The COVID-19 epidemic was a major reason for frequent admissions to hospitals. Providing effective and timely medical services can have a great impact on reducing mortality and harm to patients. Afghan immigrants, as people who live in this country, should receive medical services similar to other Iranian citizens. Considering the large population of Afghan immigrants in Kerman and the frequency of their traumas, service delivery to these immigrants is highly important.

 Considering that the improvement of the health of immigrants and all vulnerable people depends on equality and justice,^17^ there is a need for a thorough examination of differences in providing emergency health services to Afghan immigrants and Iranian citizens. To this end, the present study sought to compare services provided to trauma victims and COVID-19 patients between Iranian citizens and Afghan immigrants.

## Materials and Methods

###  Study Design and Sampling

 This cross-sectional study was conducted in Kerman, the capital of Iran’s largest province in terms of geography, with a population of about 800 000 people. To check the quality of inpatient care for Afghan immigrants during the COVID-19 period, medical services received by this group were compared with those received by Iranian patients between February 2019 and March 2021. Among all the reasons for hospitalization, trauma victims and COVID-19 patients were selected as samples. The length of hospitalization (LoH), intensive care unit (ICU) admission rate, and death rate were the most important variables that underwent investigation. Data were collected from two large hospitals in Kerman, namely, Afzalipour (COVID-19 patients) and Shahid Bahonar (trauma victims) Hospitals.

 Afzalipour Kerman hospital, with a capacity of about 700 beds, is one of the largest hospitals in southeastern Iran. It served as the central hospital in the southeast of the country during the COVID-19 outbreak. Shahid Bahonar hospital, with a capacity of about 350 beds, is the first and largest trauma center in the southeast of the country and admits and treats patients injured in accidents. Thus, a search in the databanks of these two hospitals can provide a good estimate of patient data in the southeast of the country.

## Variables

 To compare the quality of healthcare services provided for Iranian patients and Afghan nationals in both hospitals, LoH, admissions to the ICU, and the number of deaths were taken as response variables. Age, gender, and nationality of the victims of trauma admitted to Shahid Bahonar hospital were used as the independent variables. The independent variables related to COVID-19 patients admitted to Afzalipour Hospital were age, gender, nationality, and number of risk factors and chronic diseases (NRFCD). The risk factors assessed in this study were smoking and opium use, and the chronic diseases were diabetes, high blood pressure, asthma, and other respiratory disorders, neurological disorders, heart diseases, kidney diseases, cancer, blood diseases, immunodeficiency diseases, *human immunodeficiency virus*/acquired immunodeficiency syndrome, and liver diseases.

 Given the high NRFCD, the effect of each of them was not assessed in this study; it was only sought to adjust their effects. Thus, the number of chronic diseases and risk factors for each patient was considered an independent variable in the model named NRFCD.

###  Data Cleaning 

 Following the objectives of the study, some revisions were made to the data. Hence, those patients with COVID-19 or trauma who had been admitted to the hospital as outpatients or had been hospitalized temporarily (less than 6 hours) were excluded from the study.

###  Trauma Patients

 The trauma patients with incomplete medical records were excluded from the study. The number of these patients was very small (around 10 people). Accordingly, the data for 8080 trauma patients admitted to Shahid Bahonar Hospital in Kerman were analyzed in the study.

###  COVID-19 Patients 

 The COVID-19 patients whose nationality was not specified were excluded from the study. Given that the focus of the study was on adult patients with COVID-19, patients under 14 years of age were not included in the study. The data of 8686 patients with positive polymerase chain reaction tests who were admitted to Afzalipour hospital in Kerman were analyzed in this study.

###  Statistical Analysis

 The results are presented as medians ± interquartile range (IQR) and means ± standard deviations (SD) for quantitative variables, as well as frequencies and percentages for qualitative variables. Considering that LoH did not follow a normal distribution, quantile regression analysis was used to investigate the relationship between the variables and LoH. The index utilized in quantile regression analysis to compare groups was the median. Multiple logistic regression analyses were also run to evaluate the relationship between research variables and the transfer of the patient to the ICU.

 Multiple Cox regression analysis was applied to examine the relationship between the research variables (nationality and the like) and the patient’s survival time. Univariate regression was performed, and variables with *P* values less than 0.2 were eligible for the multiple regression model. Because nationality was taken as the main variable, it was considered a fixed variable with any *P* value in the multiple analysis.

 All statistical analyses were performed in SPSS software (version 26.0) for Windows (SPSS Inc., Chicago, IL). A *P* value less than 0.05 was considered statistically significant.

## Results

###  Descriptive Statistics

 Out of 8080 patients admitted to the hospital due to trauma, 82.7% were men, 91.8% were Iranian, and about 8.2% were Afghan nationals. Overall, 17% of patients were transferred to the ICU, and 2.8% of patients died during this period.

 The median age of the patients admitted to the ICU and the patients who died was 30 and 40 years, respectively. The median LoH for men and women was equal to 2 days. The median LoH was 2 and 3 days for Iranian nationals and Afghan nationals, respectively. Among trauma victims who needed ICU services, 9.6% were Afghan nationals. Moreover, 11% of the total deaths occurred among Afghan nationals.

 Out of a total of 8686 COVID-19 patients with positive polymerase chain reaction tests, 51.9% were men, 97.4% were Iranian citizens, and 2.6% were Afghan nationals. About 12.8% of the patients were transferred to the ICU, and 16.3% of them died. In addition, 56.5% of the patients had no risk factors or chronic disease (NRFCD = 0), and 15% of the patients had been injected with COVID-19 vaccines. The median age of the patients admitted to the ICU and the patients who died was 65 and 67.5 years, respectively. Patients who were admitted to the ICU or died had more risk factors. The median LoH for Afghans and Iranians was 5 days. Approximately 3% of the patients with COVID-19 who needed ICU services were Afghan nationals. Moreover, 3.2% of the total deaths due to COVID-19 were found in Afghan nationals. The median LoH of patients who received the COVID-19 vaccine was almost one day longer than that of the patients who did not receive the vaccine. Further, about 18% of all patients who were admitted to the ICU or died had received the COVID-19 vaccine (Table 1).

**Table 1:** Descriptive Statistics of Study Variables

**Variables and Setting**			**LOH** **Median±[IQR: (Q3-Q1)]** **n (%)**	**Non-ICU care** **Median±[IQR: (Q3-Q1)]** **n (%)**	**ICU Care** **Median±[IQR: (Q3-Q1)]** **n (%)**	**Alive** **Median±[IQR: (Q3-Q1)]** **n (%)**	**Death** **Median±[IQR: (Q3-Q1)]** **n (%)**
Trauma	Age		—	26.00 ± [21.00 (39.00‒18.00)]	30.00 ± [26.00 (45.00‒19.00)]	26.00 ± [21.00 (39.00‒18.00)]	40.00 ± [41.00 (61.00‒20.00)]
	Gender	Female	2.00 ± [4.00 (5.00‒1.00)] 1401 (17.30)	1140 (17.00)	261 (19.10)	1367 (17.40)	34 (15.00)
		Male	2.00 ± [4.00 (5.00‒1.00)] 6679 (82.70)	5570 (83.00)	1109 (80.90)	6486 (82.60)	193 (85.00)
	Nationality	AFG	3.00 ± [4.00 (5.00‒1.00)] 665 (8.20)	533 (7.90)	132 (9.60)	640 (8.10)	25 (11.00)
		IRI	2.00 ± [4.00 (5.00‒1.00)] 7415 (91.80)	6177 (92.10)	1238 (90.40)	7213 (91.90)	202 (89.00)
COVID-19	Age		—	53.00 ± [27.00 (66‒39)]	65.00 ± [25.00 (76.00‒51.00)]	52.00 ± [27.00 (65.00‒38.00)]	67.50 ± [23.00 (78.00‒55.00)]
	NRFCD		—	0.0 (0.0, 1.0)	1.0 (0.0, 2.0)	0.0 (0.0, 1.0)	1.0 (0.0, 2.0)
	Gender	Female	6.00 ± [5.00 (9.00‒4.00)] 4177 (48.10)	3688 (48.70)	489 (44.10)	3775 (49.2)	602 (42.4)
		Male	5.00 ± [6.00 (9.00‒3.00)] 4509 (51.90)	2889 (51.30)	620 (55.90)	3691 (50.8)	818 (57.6)
	Nationality	AFG	5.00 ± [5.00 (8.00‒3.00)] 226 (2.60)	193 (2.50)	33 (3.00)	181 (2.5)	45 (3.20)
		IRI	5.00 ± [6.00 (9.00‒3.00)] 8460 (97.40)	7384 (97.50)	1076 (97.00)	7085 (97.5)	1375 (96.8)
	Vaccine	No	5.00 ± [5.00 (8.00‒3.00)] 4723 (85.00)	4172 (85.50)	551 (81.30)	4041 (85.60)	682 (81.80)
		Yes	6.00 ± [5.00 (9.00‒4.00)] 834 (15.00)	707 (14.50)	127 (18.70)	682 (14.40)	152 (18.20)

*Note*. IQR, Interquartile range; LOH: Length of hospitalization; IQR: Interquartile range; ICU: Intensive care unit; AFG: Afghan; IRI: Iranian; NRFCD: Number of risk factors and chronic diseases.

**Figure 1:**
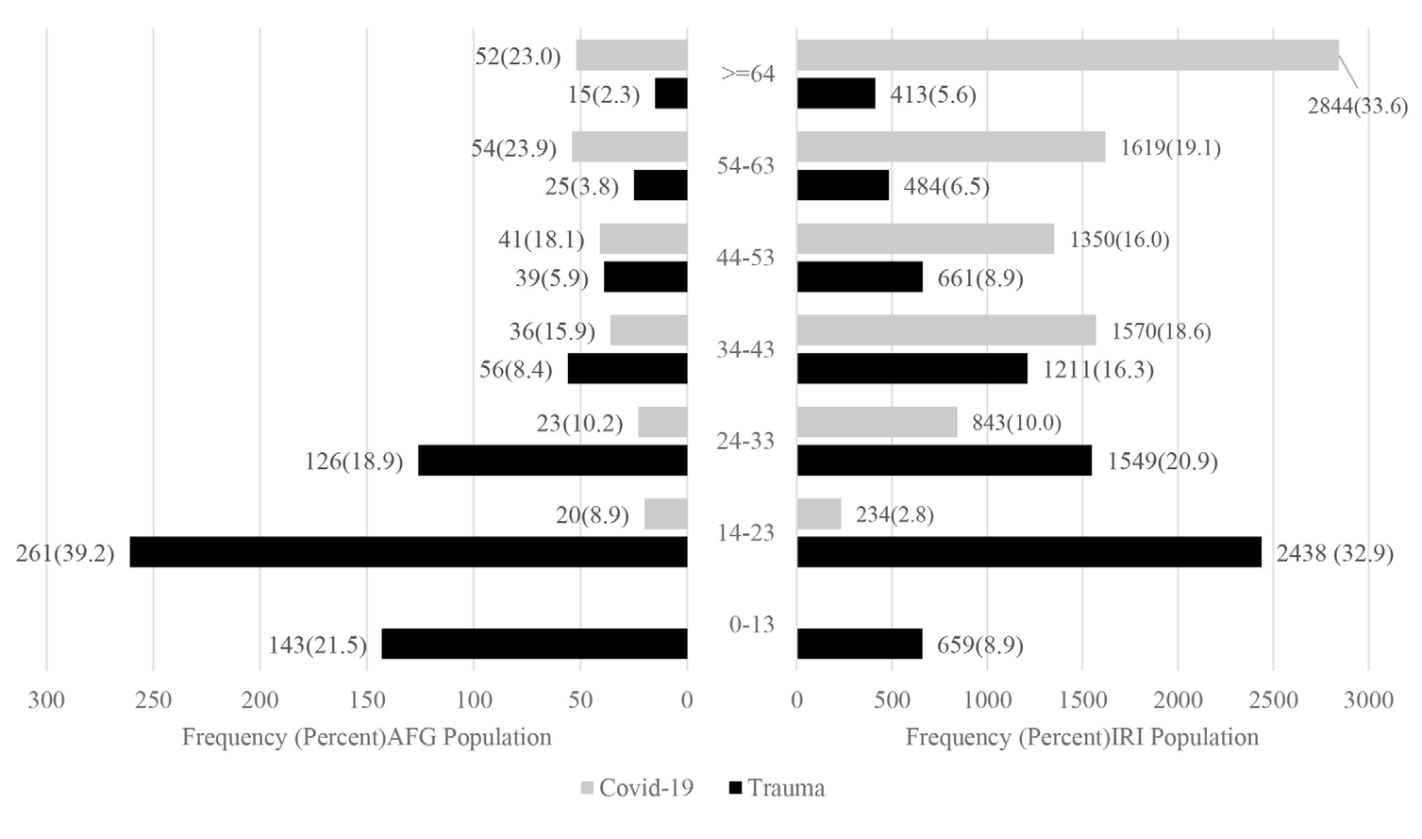
Frequency Distribution of Trauma and COVID-19 Patients by Age Group and Nationality

 Most of the trauma patients from both nationalities were in the age group of 15‒25 years. Nearly half of the trauma patients from both nationalities were in the young age group, 15‒34 years old (Figure 1).

 The highest and lowest numbers of COVID-19 patients from both nationalities were in the age groups of > 54 years and 14‒23 years, respectively (Figure 1).

###  The Relationship between Variables and Length of Hospitalization

####  Trauma Patients 

 Multiple quantile regression revealed that patients’ age and nationality had a significant effect on LoH. The median LoH was 0.34 days (approximately 8.16 hours) more for Afghan nationals than for Iranian citizens. Moreover, for every one-year increase in age, the median LoH increased by 0.02 days (approximately 29 minutes, Table 2).

**Table 2:** Quantile Regression Analysis for the Relationship of Variables With LoH in Traumas and COVID-19 Patients

**Variables and Setting**			**Univariate Regression**		**Multiple Quantile Regression**	
			**Coefficient (95% CI)**	***P*** ** Value**	**Coefficient (95% CI)**	***P*** ** Value**
Trauma	Age		0.02 (0.01, 0.021)	< 0.001	0.02 (0.01, 0.03)	< 0.001
	Gender	Female	REF	—	—	—
		Male	0.00 (-0.13, 0.13)	1.00	—	—
	Nationality	IRI	REF	—	REF	—
		AFG	1.00 (0.82, 1.19)	< 0.001	0.34 (0.13, 0.56)	< 0.01
COVID-19	Age		0.02 (0.01, 0.03)	< 0.001	0.02 (0.01, 0.03)	< 0.001
	NRFCD		0.33 (0.26, 0.40)	< 0.001	0.14 (0.03, 0.24)	0.01
	Gender	Female	REF	—	REF	—
		Male	-1.00 (-0.80, -1.20)	< 0.001	-0.08 (-0.29, 0.14)	0.48
	Nationality	IRI	REF	—	—	—
		AFG	0.00 (-0.31, 0.31)	1.00	-0.65 (-1.31, 0.002)	0.06
	Vaccine	No	REF	—	REF	—
		Yes	1.00 (0.68, 1.32)	< 0.001	0.10 (-0.21, 0.40)	0.53

*Note*. Confidence intervals;LOH: Length of hospitalization; IQR: Interquartile range; ICU: Intensive care unit; AFG: Afghan; IRI: Iranian; NRFCD: Number of risk factors and chronic diseases.

####  COVID-19 Patients 

 Multiple quantile regression indicated that patients’ age and sum of risk factors had a significant effect on the median LoH. In the presence of other investigated factors, for every one-year increase in age, the median LoH increased by 0.02 days (about 29 minutes).

 For each underlying disease or risk factor increase in the patient, LoH increased by 0.14 days (approximately 3.36 hours). Patients’ gender, nationality, and vaccine status, however, did not have a significant effect on the median LoH (Table 2). The results related to the impact of individual risk factors on LoH are provided in Table S1 of the supplementary material.

###  The Relationship between Research Variables with Admission to the Intensive Care Unit and Death of Patients

####  Admission to the Intensive Care Unit

#####  Trauma Patients

 The results of multiple logistic regression analysis showed that the patients’ nationality and age could significantly affect their admission to the ICU. The chance of Afghan nationals being admitted to the ICU was 38% higher than that of Iranian citizens. Moreover, for every one-year increase in age, the chance of receiving ICU care increased by 1% (Table 3).

**Table 3:** Logistic Regression Analysis for ICU Admission in Trauma and COVID-19 Patients

**Variables and Setting**		**Univariate Logistic Regression**			**Multiple Logistic Regression**	
		**OR (95% CI)**		***P *** **Value**	**OR (95% CI)**	***P *** **Value**
Trauma	Age	1.01 (1.0, 1.02)		< 0.001	1.01 (1.00, 1.02)	< 0.001
	Gender	Female	REF	—	REF	—
		Male	0.87 (0.75, 1.01)	0.066	0.94 (0.80, 1.09)	0.39
	Nationality	IRI	REF		REF	—
		AFG	1.24 (1.01, 1.51)	0.04	1.38 (1.12, 1.69)	< 0.01
COVID-19	Age	1.031 (1.03, 1.035)		< 0.001	1.03 (1.02, 1.04)	< 0.001
	NRFCD	1.24 (1.18, 1.30)		< 0.001	1.11 (1.04, 1.19)	< 0.01
	Gender	Female	REF	—	REF	—
		Male	1.20 (1.06, 1.36)	0.004	1.17 (1.00, 1.38)	0.06
	Nationality	IRI	REF	—	—	—
		AFG	1.17 (0.81, 1.71)	0.40	1.30 (0.80, 2.11)	0.29
	Vaccine	No	REF	—	REF	—
		Yes	1.36 (1.10, 1.68)	< 0.01	1.08 (0.87, 1.34)	0.42

*Note*. OR, odds ratio; CI, Confidence interval; LOH: Length of hospitalization; IQR: Interquartile range; ICU: Intensive care unit; AFG: Afghan; IRI: Iranian; NRFCD: Number of risk factors and chronic diseases.

#####  COVID-19 Patients 

 The results of multiple regression analysis demonstrated that only age and NRFCD were effective on admission to the ICU; neither gender, nationality, nor vaccination status had a significant effect on admission to the ICU. For every one-year increase in patients’ age, the chance of being admitted to the ICU increased by 3%. Furthermore, for each underlying disease or risk factor increase in the patient, the chance of being admitted to the ICU increased by 11% (Table 3). The details related to the impact of each risk factor on being admitted to the ICU are presented in Table S2 of the supplementary material.

####  Patient’s Death Rate 

#####  Trauma Patients 

**Figure 2:**
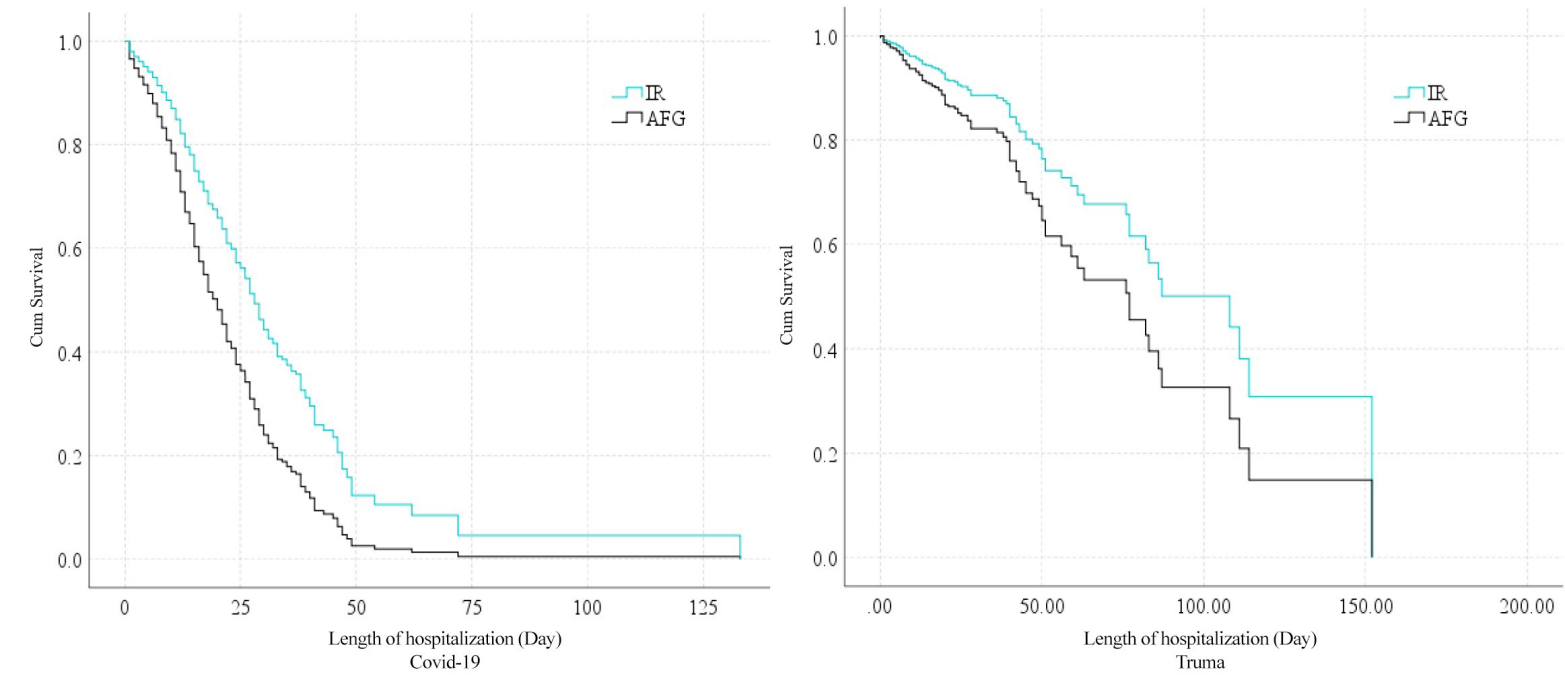
Survival Rate Among Patients Referred to Two Emergency Centers

 The survival rate according to nationality was reported in Figure 2. The survival rate of Iranian nationality was higher than that of Afghan nationality. The mean (95% CI) survival time of patients with Afghan and Iranian nationalities was 109.36 (85.70, 133.03) and 82.01 (70.75, 93.28) days, respectively. The results of multiple Cox regression analysis confirmed that age and nationality had a significant effect on the deaths of patients. It was also found that the hazard of death was 60% higher for Afghan nationals than for Iranians. Moreover, the hazard of death increased by 3% for every one-year increase in age (Table 4).

**Table 4:** Survival Analysis for the Death Rate in Trauma and COVID-19 Patients

**Variables and Setting**			**Univariate Cox Regression**		**Multiple Cox Regression**	
			**HR (95% CI)**	***P *** **Value**	**HR (95% CI)**	***P *** **Value**
Trauma	Age		1.02 (1.01, 1.03)	< 0.001	1.03 (1.01, 1.04)	< 0.001
	Gender	Female	REF	—	—	—
		Male	1.28 (0.87, 1.89)	0.21	—	—
	Nationality	IRI	REF	—	REF	—
		AFG	1.33 (0.86, 2.05)	0.20	1.60 (1.03, 2.49)	0.04
COVID-19	Age		1.03 (1.02, 1.04)	< 0.001	1.03 (1.02, 1.04)	< 0.001
	NRFCD		1.18 (1.00, 1.40)	0.049	0.91 (0.70, 1.19)	0.51
	Gender	Female	REF	—	REF	—
		Male	1.20 (1.08, 1.33)	0.001	1.19 (1.04, 1.37)	< 0.001
	Nationality	IRI	REF	—	REF	—
		AFG	1.32 (0.98, 1.78)	0.07	1.36 (0.92, 2.00)	0.12
	Vaccine	No	REF	—	REF	—
		Yes	1.15 (0.97, 1.38)	0.11	1.16 (0.97, 1.39)	0.10

*Note*. HR, hazard ratio; CI, Confidence interval; LOH: Length of hospitalization; IQR: Interquartile range; AFG: Afghan; IRI: Iranian; NRFCD: Number of risk factors and chronic diseases.

#####  Coronavirus Disease Patients 

 The survival rate according to nationality is reported in Figure 2. This factor was not different between Iranian and Afghan nationalities.

 The mean (95% CI) survival time of Afghan patients and Iranian patients was 22.86 (18.56, 27.17) and 30.21 (26.75, 33.68) days, respectively.

 The results of multiple Cox regression analysis revealed that patients’ age and gender could significantly affect their deaths. As the age of the patient increased by one year, the hazard of death due to COVID-19 increased by 3%. The hazard of death was 19% higher in men than in women. There was no significant difference in the deaths of patients of Afghan and Iranian nationalities (Table 4). The data associated with the impact of each risk factor on the death of patients are listed in Table S3 of the supplementary material.

## Discussion

 This study compared the medical services provided to Iranian citizens and Afghan immigrants admitted to two hospitals in Kerman because of trauma and COVID-19. The results showed that most of the trauma patients (Iranian citizens and Afghan immigrants) were men (83%). More than half of the Afghan and Iranian nationals who had trauma were in the age group of 15‒34 years. Unfortunately, 17% of the patients were transferred to the ICU, and 2.8% of them died. Meanwhile, the median age of those who died and those admitted to the ICU was 14 and 4 years higher than other trauma victims, respectively. These findings imply that a large number of trauma victims were young people. While other incidents aside from accidents can also result in trauma, traffic accidents remain the most significant cause and a prominent contributor to the referral of trauma patients. Numerous studies in this field have established that individuals in the young age group are prone to sustaining trauma injuries at a higher rate than other age groups.^18^

 For example, a review study in Iran demonstrated that the mean age of people involved in traffic accidents was 30 years, and more than three-quarters of the patients were men.^19^ Similar to their counterparts in most low-income countries (e.g., Afghanistan^20^) and middle-income countries compared to high-income countries, Iranian men seem to be more vulnerable to injuries because of Iran’s specific socio-cultural conditions (more driving, more occupational threats, injuries from violence, and the like).^19,21^ However, as people’s age increases, they are more likely to undergo more severe outcomes, such as hospitalization and death. The findings of a study in Taiwan revealed that, compared to other adults injured in traffic accidents, elderly trauma patients had higher injury severity, less favorable outcomes, and a higher rate of admission to the ICU.^22^

 Based on the findings, the median LoH of trauma patients increased with increasing age. Similarly, many studies have shown that older adults were hospitalized for a longer period in the hospital and ICU than other age groups and had higher mortality.^22^ In general, in-hospital mortality was higher in pedestrians and elderly motorcyclists compared to the younger elderly and other victims.^23^

 The data in this study confirmed that age and Afghan nationality increased the median LoH due to traumas by 0.48–8.16 hours, respectively. Moreover, the odds of admission to the ICU and the hazard of death for Afghan nationals were 1.38 times and 1.60 times higher than for Iranians, respectively. Although traffic accidents are an important health challenge in Iran, the evidence shows that certain determining factors make immigrants in different parts of the world more prone to injury. In a study in Canada, due to differences in road management culture, immigrant students had 1.3 times more bicycle injuries than native students.^24^ Immigrants from Ghana, Iraq, Somalia, Turkey, and Ethiopia had the highest number of emergency room visits due to motor vehicle accident injuries.^24^ Furthermore, a study in Canada reported that the rate of hospitalization in immigrants due to unintentional accidents was higher than that of native people. Immigrants had higher injury rates for most causes, including motor vehicle injuries, poisoning, and suffocation.^25^

 Immigrants are more vulnerable to accidents for various reasons, such as climatic, cultural-economic, and income differences.^26^ It also seems that factors such as language problems, concerns about family in the country of origin, attitudes toward traffic laws, and driving behavior reflected in the number of fines for road traffic violations can help explain the difference in the risk of road accidents.^26^

 Even though an increasing number of immigrants and refugees are currently elderly, disabled, or even minors, in general, the majority of refugees are young.^27^ Thus, it seems that the difference in risk-taking behavior between the immigrant population and the host population is also effective in observing the more severe consequences of accidents. Moreover, another potential factor is the different threshold for seeking medical care for symptoms of illness and injury.^28^ One possible explanation is the so-called healthy immigrant effect, which assumes that because of the difficult process of immigration, immigrants have a health advantage over the domestic-born, which vanishes with increased length of residency. However, most studies have focused on physical health, and less attention has been paid to immigrants’ mental health.^29,30^ Thus, it seems that only more severe cases seek healthcare services. However, according to the evidence, immigrants and refugees often have a less or equal desire to use health services compared to the native population.^31^ The willingness to receive or not receive health services is different in immigrant groups according to the country of origin, the reason for migration, legal status, LoH, socio-demographic variables, and people’s needs.^31^ It seems that reasons such as fear of reporting their immigration status to law enforcement, organizational discrimination, poverty, and low literacy are also effective factors in reducing their willingness to receive health facilities.^7^ Nevertheless, factors such as discrimination and weak social support also make refugees more susceptible to injuries.^32^ Free primary healthcare for immigrants in Iran showed its high efficacy during the COVID-19 pandemic.

 The present study revealed that the highest and lowest numbers of COVID-19 patients from both nationalities were in the age group of more than 54 years and 14‒23 years, respectively. In addition, the male gender increases the hazard of dying from COVID-19 by 36% (1.4). Much other evidence confirms these findings. Given the prevalence of many diseases such as high blood pressure, chronic respiratory diseases, diabetes, cardiovascular diseases, and the like, the older age group experienced more and more severe cases of COVID-19.^33,34^ Moreover, men were more affected than women and had a higher chance of death.^35,36^ According to the results of a review study, the chance of death in men was 91.3 times that of women.^37^

 The median LoH from COVID-19 increased with an increase in age and the NRFCD. However, gender, nationality (Iranian or Afghan), and COVID-19 vaccination status did not have a significant effect on the median LoH. It seems that one of the reasons for this equality and lack of difference between the two nationalities was the adequate access to healthcare services during the COVID-19 outbreak. Paying attention to the health of immigrants in Iran has always been considered a principle of human rights. Public insurance services have provided Afghan refugees, similar to Iranians, access to secondary and tertiary healthcare. Nevertheless, it seems that refugees and immigrants in different parts of the world faced many problems during the COVID-19 pandemic, and various factors can make them vulnerable to the direct and indirect effects of COVID-19.

 In many countries, immigrants, especially those with irregular status or short-term visas, do not have equal access to healthcare as citizens and may not be covered for treatment for COVID-19.^38-40^ Even in cases where they are entitled to relevant services, language barriers, limited knowledge of host country conditions, or prioritization of citizens may result in insufficient access to healthcare.^41^ Immigrants also have less access to general practitioners, so they tend to have limited access to preventive care and instead rely on hospitals,^41^ which is both more difficult and dangerous as emergency services are saturated with COVID-19 patients. Additionally, undocumented immigrants may fear being reported to immigration authorities and deported if they seek help, which may reduce their willingness to seek screening, testing, contact tracing, or treatment.^42,43^ In Iran, illegal immigrants do not have insurance coverage.

## Conclusion

 The findings of this study showed that more Afghan nationals injured in accidents were admitted to the ICU or died. It seems that patients with more serious injuries refer to the hospital, but this was not the case with COVID-19 patients. It seems that with the help of the government to organize illegal immigrants and insure them, Afghan immigrants with less severe injuries also visit hospitals. Most young people of both nationalities had physical traumas. Thus, politicians need to help reduce the number of young people injured by developing educational programs and awareness-raising activities. Finally, following the findings of this study, it seems that justice in treatment has been fully established for Afghan patients, similar to Iranian patients.

## Limitations and Strengths

 This study was conducted with several limitations. Unfortunately, the number of years the refugees were residing in Iran, an effective factor, was not addressed in this study; some Afghan immigrants came to Iran many years ago, and others entered Iran in the last one or two years due to the presence of the Taliban. The severity of the COVID-19 disease and the traumas were not investigated in this study. Furthermore, we did not have any information about the overall age-gender composition of Afghan immigrants. However, one of the strengths of this study was its focus on the age groups of the patients. Age is effective in both cases of traffic accidents and COVID-19 cases, and with the separation of age groups, the impact of age was statistically controlled in the data analysis to make a correct comparison between Afghan nationals and citizens in Kerman.

## Supplementary File

Supplementary file contains Table S1-S3.

## References

[1] Seddighi H, Naseh M, Rafieifar M, Ilea P (2024). Education of Afghan refugee children in Iran: a structured review of policies. Child Soc.

[2] Siavoshi S (2024). Afghans in Iran: the state and the working of immigration policies. Br J Middle East Stud.

[3] UNHCR. Refugees in Iran. UNHCR; 2022.

[4] UNHCR. Refugees in Kerman, Iran. UNHCR; 2022.

[5] World Health Organization (WHO). World Report on the Health of Refugees and Migrants. WHO; 2022.

[6] Langlois EV, Haines A, Tomson G, Ghaffar A (2016). Refugees: towards better access to health-care services. Lancet.

[7] Mangrio E, Sjögren Forss K (2017). Refugees’ experiences of healthcare in the host country: a scoping review. BMC Health Serv Res.

[8] UNHCR. UNHCR Iran: Operational Update. UNHCR; 2021.

[9] Shamsi Gooshki E, Rezaei R, Wild V (2016). Migrants’ health in Iran from the perspective of social justice: a systematic literature review. Arch Iran Med.

[10] Hosseini Divkolaye NS, Burkle FM Jr. The enduring health challenges of Afghan immigrants and refugees in Iran: a systematic review. PLoS Curr 2017;9. 10.1371/currents.dis.449b4c549951e359363a90a7f4cf8fc4. PMC5554007 28856065

[11] Verma AK, Prakash S (2020). Impact of COVID-19 on environment and society. J Glob Biosci.

[12] Worldometer. COVID - Coronavirus Statistics. Worldometer; 2022.

[13] Nakhaeizadeh M, Eybpoosh S, Jahani Y, Ahmadi Gohari M, Haghdoost AA, White L (2022). Impact of non-pharmaceutical interventions on the control of COVID-19 in Iran: a mathematical modeling study. Int J Health Policy Manag.

[14] Nakhaeizadeh M, Chegeni M, Adhami M, Sharifi H, Ahmadi Gohari M, Iranpour A (2022). Estimating the number of COVID-19 cases and impact of new COVID-19 variants and vaccination on the population in Kerman, Iran: a mathematical modeling study. Comput Math Methods Med.

[15] Ahmadi Gohari M, Chegeni M, Haghdoost AA, Mirzaee F, White L, Kostoulas P (2022). Excess deaths during the COVID-19 pandemic in Iran. Infect Dis (Lond).

[16] Iranian legal Medical Organization, Accident in Iran. 2021. Available from: https://en.lmo.ir/.

[17] Kiani MM, Khanjankhani K, Takbiri A, Takian A (2021). Refugees and sustainable health development in Iran. Arch Iran Med.

[18] Adib-Hajbaghery M, Maghaminejad F (2014). Epidemiology of patients with multiple trauma and the quality of their prehospital respiration management in Kashan, Iran: six months assessment. Arch Trauma Res.

[19] Azami-Aghdash S, Sadeghi-Bazargani H, Shabaninejad H, Abolghasem Gorji H (2017). Injury epidemiology in Iran: a systematic review. J Inj Violence Res.

[20] World Health Organization (WHO). Global Status Report on Road Safety 2018. WHO; 2018.

[21] Centers for Disease Control and Prevention (CDC). National Hospital Discharge Survey. CDC; 2022.

[22] Hsieh CH, Liu HT, Hsu SY, Hsieh HY, Chen YC (2017). Motorcycle-related hospitalizations of the elderly. Biomed J.

[23] Etehad H, Yousefzadeh-Chabok S, Davoudi-Kiakalaye A, Moghadam Dehnadi A, Hemati H, Mohtasham-Amiri Z (2015). Impact of road traffic accidents on the elderly. Arch Gerontol Geriatr.

[24] Government of Alberta. Immigrant Health in Alberta. Government of Alberta; 2011.

[25] Saunders NR, Macpherson A, Guan J, Guttmann A (2018). Unintentional injuries among refugee and immigrant children and youth in Ontario, Canada: a population-based cross-sectional study. Inj Prev.

[26] Assum T, Nordbakke S. Accidents risk and road safety among immigrants in Norway. In: 16th International Conference Road Safety on Four Continents. Beijing, China: Swedish National Road and Transport Research Institute; 2013.

[27] World Health Organization (WHO). Report on the Health of Refugees and Migrants in the WHO European Region: No Public Health Without Refugee and Migrant Health. WHO; 2018.

[28] Ohm E, Holvik K, Kjøllesdal MKR, Madsen C (2020). Health care utilisation for treatment of injuries among immigrants in Norway: a nationwide register linkage study. Inj Epidemiol.

[29] McDonald JT, Kennedy S (2004). Insights into the ‘healthy immigrant effect’: health status and health service use of immigrants to Canada. Soc Sci Med.

[30] Elshahat S, Moffat T, Newbold KB (2022). Understanding the healthy immigrant effect in the context of mental health challenges: a systematic critical review. J Immigr Minor Health.

[31] Sarría-Santamera A, Hijas-Gómez AI, Carmona R, Gimeno-Feliú LA (2016). A systematic review of the use of health services by immigrants and native populations. Public Health Rev.

[32] Borsch AS, de Montgomery CJ, Gauffin K, Eide K, Heikkilä E, Smith Jervelund S (2019). Health, education and employment outcomes in young refugees in the Nordic countries: a systematic review. Scand J Public Health.

[33] Yang J, Zheng Y, Gou X, Pu K, Chen Z, Guo Q (2020). Prevalence of comorbidities and its effects in patients infected with SARS-CoV-2: a systematic review and meta-analysis. Int J Infect Dis.

[34] Wang T, Du Z, Zhu F, Cao Z, An Y, Gao Y (2020). Comorbidities and multi-organ injuries in the treatment of COVID-19. Lancet.

[35] Jin JM, Bai P, He W, Wu F, Liu XF, Han DM (2020). Gender differences in patients with COVID-19: focus on severity and mortality. Front Public Health.

[36] Li LQ, Huang T, Wang YQ, Wang ZP, Liang Y, Huang TB (2020). COVID-19 patients’ clinical characteristics, discharge rate, and fatality rate of meta-analysis. J Med Virol.

[37] Peckham H, de Gruijter NM, Raine C, Radziszewska A, Ciurtin C, Wedderburn LR (2020). Male sex identified by global COVID-19 meta-analysis as a risk factor for death and ITU admission. Nat Commun.

[38] Collins FL. Caring for 300,000 Temporary Migrants in New Zealand is a Crucial Missing Link in Our Coronavirus Response. The Conversation; 2020. p. 26.

[39] Kaiser Family Foundation (KFF). Health Coverage of Immigrants. Washington, DC: KFF; 2020.

[40] Vearey J, Hui C, Wickramage K (2020). Migration and health: current issues, governance and knowledge gaps. World Migration Report.

[41] Kohn D. Nearly half of US medical care comes from emergency rooms. In: Figures Are Even Higher for Minorities and Women. University of Maryland School of Medicine; 2017.

[42] D’ignoti S. How Coronavirus Hits Migrants and Asylum Seekers in Italy. The New Humanitarian; 2020.

[43] Jordan M. We’re Petrified’: Immigrants Afraid to Seek Medical Care for Coronavirus. New York Times; 2020. p. 18.

