# Supplementary material for: Comparing the Emergency Care of Iranian and Afghan Patients During the COVID-19 Pandemic
Source: Arch Iran Med. 2024 Mar 1;27(3):142–50. doi: 10.34172/aim.2024.22 (PMC11097311; doi:10.34172/aim.2024.22)
Supplement: Supplementary file 1 — Supplementary file contains Table S1-S3. [file aim-27-142-s001.pdf]

Arch Iran Med. March 2024;27(3):142-S1

10.34172/aim.2024.22

Supplementary Material

Table S1. Quantile regression analysis for the relationship of variables with LoH in trauma and Covid-19 patients

Variables & Setting			Univariate Quantile Regression		Multiple Quantile Regression	
			**Coefficient (95% CI)***	**P-value**	**Coefficient (95% CI)***	***P*-Value**
Trauma	Age		0.02 (0.01, 0.03)	<0.001	0.02 (0.01, 0.03)	<0.001
	Gender	Female	REF		–	–
		Male	0.00 (-0.13, 0.13)	1.00	–	–
	Nationality	IRI	REF		REF	–
		AFG	1.00 (0.82, 1.19)	<0.001	0.34 (0.13, 0.56)	<0.01
Covid-19	Age		0.02 (0.01, 0.03)	<0.001	0.01 (0.001, 0.02)	<0.001
	Gender	Male	REF	–	REF	–
		Female	1.00 (0.80, 1.20)	<0.001	0.03 (-0.19, 0.24)	0.81
	Nationality	IRI	REF	–	–	–
		AFG	0.00 (-0.31, 0.31)	1.00	-0.65 (-1.31, 0.002)	0.051
	Vaccine	NO	REF	–	REF	–
		Yes	1.00 (0.68, 1.32)	<0.001	0.07 (-0.24, 0.37)	0.67
	Diabetes	NO	REF	–	REF	–
		Yes	1.00 (0.85, 1.15)	<0.001	0.71 (0.30, 1.11)	<0.01
	High blood pressure	NO	REF	–	REF	–
		Yes	1.00 (0.86, 1.14)	<0.001	-0.08 (-0.46, 0.30)	0.68
	Asthma	NO	REF	–	–	–
		Yes	0.00 (-0.55, 0.55)	1.00	–	–
	Other respiratory disorders except for asthma	NO	REF	–	REF	–
		Yes	1.00 (0.81, 1.19)	<0.001	0.59 (0.08, 1.09)	0.02
	Neurological disorders	NO	REF	–	REF	–
		Yes	1.00 (0.64. 1.36)	<0.001	0.36 (-0.62, 1.34)	0.47
	Heart diseases	NO	REF	–	REF	–
		Yes	1.00 (0.83, 1.17)	<0.001	0.42 (-0.04, 0.88)	0.08
	Kidney diseases	NO	REF	–	REF	–
		Yes	1.00 (0.73, 1.27)	<0.001	0.49 (-0.20, 1.19)	0.16
	Cancer	NO	REF	–	REF	–
		Yes	1.00 (0.63, 1.37)	<0.001	0.66 (-0.19, 1.52)	0.13
	Immunodeficienc y diseases	NO	REF	–	REF	–
		Yes	1.00 (0.12, 1.88)	0.03	1.25 (-1.15, 3.65)	0.31
	HIV/Aids	NO	REF	–	REF	–
		Yes	5.00 (3.53, 6.47)	<0.001	4.87 (1.32, 8.42)	<0.01
	Liver diseases	NO	REF	–	REF	–
		Yes	1.00 (0.50, 1.50)	<0.001	-0.17 (-1.52, 1.18)	0.80
	smoking	NO	REF	–	REF	–
		Yes	0.00 (-0.22, 0.22)	1.00	–	–
	Opium use	NO	REF	–	REF	–
		Yes	0.00 (-0.15, 0.15)	1.00	–	–

*Confidence Intervals

Table S2. Logistic regression analysis for ICU admission in trauma and Covid-19 patients

Variables & Setting			Univariate Logistic Regression		Multiple Logistic Regression	
			**OR (95% CI)***	***P*-Value**	**OR (95% CI)***	**P-Value**
Trauma	Age		1.01 (1.0, 1.02)	<0.001	1.01 (1.00,1.02)	<0.001
	Gender	Female	REF	–	REF	–
		Male	0.87 (0.75, 1.01)	0.066	0.94 (0.80, 1.01)	0.39
	Nationality	IRI	REF		REF	–
		AFG	1.24 (1.01, 1.51)	0.04	1.38 (1.12, 1.69)	<0.01
Covid-19	Age		1.03 (1.03, 1.04)	<0.001	1.03 (1.02, 1.04)	<0.001
	Gender	Female	REF	–	–	–
		Male	1.20 (1.06, 1.4)	0.004	1.19 (1.01, 1.40)	0.04
	Nationality	IRI	REF	–	–	–
		AFG	1.17 (0.81, 1.71)	0.40	1.28 (0.79, 2.09)	0.31
	Vaccine	NO	REF	–	REF	–
		Yes	1.36 (1.10, 1.68)	0.004	1.09 (0.88, 1.35)	0.44
	Diabetes	NO	REF	–	REF	–
		Yes	1.56 (1.32, 1.85)	<0.001	1.20 (0.91, 1.58)	0.18
	High blood pressure	NO	REF	–	REF	–
		Yes	1.80 (1.55, 2.10)	<0.001	1.11 (0.86, 1.44)	0.42
	Asthma	NO	REF	–	REF	–
		Yes	0.51 (0.21, 1.28)	0.15	0.26 (0.03, 1.89)	0.18
	Other respiratory disorders except for asthma	NO	REF	–	REF	–
		Yes	1.84 (1.50, 2.26)	<0.001	1.49 (1.08, 2.05)	0.01
	Neurological disorders	NO	REF	–	–	–
		Yes	1.06 (0.68, 1.64)	0.81	–	–
	Heart diseases	NO	REF	–	REF	–
		Yes	1.83 (1.52, 2.21)	<0.001	1.08 (0.8, 1.46)	0.62
	Kidney diseases	NO	REF	–	REF	–
		Yes	1.99 (1.51, 2.64)	<0.001	1.50 (0.97, 2.32)	0.07
	Cancer	NO	REF	–	REF	–
		Yes	1.97 (1.35, 2.86)	<0.001	1.43 (0.83, 2.46)	0.2
	Immunodeficiency diseases	NO	REF	–	REF	–
		Yes	2.29 (0.97, 5.39)	0.06	0.66 (0.08, 5.30)	0.7
	HIV/Aids	NO	REF	–	–	–
		Yes	0.76 (0.10, 6.00)	0.79	–	–
	Liver diseases	NO	REF	–	–	–
		Yes	1.08 (0.58, 1.99)	0.81	–	–
	smoking	NO	REF	–	–	–
		Yes	0.95 (0.71, 1.26)	0.72	–	–
	Opium use	NO	REF	–	REF	–
		Yes	1.22 (1.01, 1.46)	0.03	0.87 (0.67, 1.13)	0.31

*Confidence Intervals

Table S3. Survival analysis for the death rate in trauma and Covid-19 patients

Variables & Setting			Univariate Cox Regression		Multiple Cox Regression	
			**HR (95% CI)***	***P*-Value**	**HR (95% CI)***	***P*-Value**
Trauma	Age		1.02 (1.01, 1.03)	<0.001	1.03 (1.01,1.04)	<0.001
	Gender	Female	REF	–	–	–
		Male	1.28 (0.87, 1.89)	0.21	–	–
	Nationality	IRI	REF	–	REF	–
		AFG	1.33 (0.86, 2.05)	0.2	1.60 (1.03, 2.49)	0.04
Covid-19	Age		1.03 (1.02, 1.04)	<0.001	1.03 (1.02, 1.04)	<0.001
	Gender	Female	REF	–	REF	–
		Male	1.20 (1.08, 1.33)	0.001	1.20 (1.04, 1.38)	0.01
	Nationality	IRI	REF	–	REF	–
		AFG	1.32 (0.98, 1.78)	0.07	1.40 (0.95, 2.05)	0.09
	Vaccine	NO	REF	–	REF	–
		Yes	1.15 (0.97, 1.38)	0.11	1.14 (0.95, 1.36)	0.16
	Diabetes	NO	REF	–	REF	–
		Yes	1.25 (1.09, 1.44)	0.001	1.11 (0.88, 1.40)	0.39
	High blood pressure	NO	REF	–	REF	–
		Yes	1.34 (1.19, 1.52)	<0.001	1.14 (0.91, 1.43)	0.24
	Asthma	NO	REF	–	REF	–
		Yes	0.47 (0.21, 1.06)	0.07	0.23 (0.03, 1.63)	0.14
	Other respiratory disorders except for asthma	NO	REF	–	REF	–
		Yes	1.66 (1.42, 1.93)	<0.001	1.40 (1.09, 1.81)	<0.01
	Neurological disorders	NO	REF	–	–	–
		Yes	1.12 (0.78, 1.61)	0.54	–	–
	Heart diseases	NO	REF	–	REF	–
		Yes	1.46 (1.26, 1.70)	<0.001	0.93 (0.71, 1.21)	0.59
	Kidney diseases	NO	REF	–	REF	–
		Yes	1.39 (1.11, 1.73)	0.004	1.28 (0.92, 1.79)	0.15
	Cancer	NO	REF	–	REF	–
		Yes	1.79 (1.35, 2.38)	<0.001	1.64 (1.06, 2.53)	0.03
	Immunodeficienc y diseases	NO	REF	–	–	–
		Yes	1.01 (0.48, 2.12)	0.99	–	–
	HIV/Aids	NO	REF	–3	–	–
		Yes	0.59 (0.08, 4.18)	0.59	–	–
	Liver diseases	NO	REF	-	REF	–
		Yes	1.39 (0.89, 2.16)	0.15	1.79 (0.93, 3.47)	0.08
	smoking	NO	REF	–	–	–
		Yes	1.10 (0.89, 1.36)	0.39	–	–
	Opium use	NO	REF	–	REF	–
		Yes	1.21 (1.05, 1.40)	0.009	0.99 (0.80, 1.22)	0.91

*Hazard Ratio (Confidence Intervals)
